# Lower cognitive reappraisal capacity is related to impairments in attachment and personality structure in poly-drug use: an fMRI study

**DOI:** 10.1007/s11682-020-00414-3

**Published:** 2020-11-21

**Authors:** M. Hiebler-Ragger, C. M. Perchtold-Stefan, H. F. Unterrainer, J. Fuchshuber, K. Koschutnig, L. Nausner, H. P. Kapfhammer, I. Papousek, E. M. Weiss, A. Fink

**Affiliations:** 1 University Clinic of Psychiatry and Psychotherapeutic Medicine, Medical University, Auenbruggerplatz 31, 8036, Graz, Austria; 2 Center for Integrative Addiction Research (CIAR), Grüner Kreis Society, Simmeringer Hauptstraße 101, 1110 Vienna, Austria; 3 Institute of Psychology, University of Graz, Universitätsplatz 2, 8010 Graz, Austria; 4 Department of Religious Studies, University of Vienna, Schenkenstraße 8-10, 1010 Vienna, Austria; 5 Fachsektion Integrative Gestalttherapie, Österreichischer Arbeitskreis für Gruppentherapie und Gruppendynamik, Lenaugasse 3, 1080 Vienna, Austria; 6 Department of Medical Psychology and Psychotherapy, Medical University of Graz, Auenbruggerplatz 43, 8036, Graz, Austria; 7 Institute of Psychology, University of Innsbruck, Innrain 52f, 6020 Innsbruck, Austria

**Keywords:** Cognitive reappraisal, Polydrug use, fMRI, Attachment, Emotion regulation

## Abstract

Insecure attachment, impaired personality structure and impaired emotion regulation figure prominently in substance use disorders. While negative emotions can trigger drug-use and relapse, cognitive reappraisal may reduce emotional strain by promoting changes in perspective. In the present study, we explored behavioral and neural correlates of cognitive reappraisal in poly-drug use disorder by testing individuals’ capability to generate cognitive reappraisals for aversive events (Reappraisal Inventiveness Test). 18 inpatients with poly-drug use disorder and 16 controls completed the Adult Attachment Scale, the Emotion Regulation Questionnaire, the Brief Symptom Inventory, the Wonderlic Personnel Test, and the Operationalized Psychodynamic Diagnosis Structure Questionnaire, as well as two versions of the Reappraisal Inventiveness Test (during fMRI and outside the lab). Compared to controls, polydrug inpatients reported impaired personality structure, attachment and emotion regulation abilities. In the Reappraisal Inventiveness Test, poly-drug inpatients were less flexible and fluent in generating reappraisals for anger-eliciting situations. Corresponding to previous brain imaging evidence, cognitive reappraisal efforts of both groups were reflected in activation of left frontal regions, particularly left superior and middle frontal gyri and left supplemental motor areas. However, no group differences in neural activation patterns emerged. This suggests that despite cognitive reappraisal impairments on a behavioral level, neural reflections of these deficits in poly-drug use disorder might be more complex.

## Introduction

Impairments in the processing and regulation of emotion represent “a liability spectrum that underlies many different mental disorders” (p. 154; Kret and Ploeger 2015). However, studies regarding the relevance of specific emotion regulation strategies and their neural correlates in substance use disorders are still relatively sparse (Aldao et al. 2010).

Formed by early dysfunctional interactions between child and caregiver (e.g., Bowlby 1977), insecure attachment (Flores 2011) and an impaired personality structure (Hiebler-Ragger et al. 2016) are well-known characteristics of substance use disorders linked to impaired emotion regulation. Furthermore, with increased levels of negative emotions present in substance use disorders and negative emotional states (e.g., depression and frustration) linked to the highest rate of relapse, improving the ability to regulate emotion is well documented as being of integral importance for the treatment of substance use disorders (Larimer et al. 1999).

### Attachment and personality structure

Emotions and their regulation have been in the focus of attachment theory since its inception and a secure attachment is regarded as highly important for the ability to regulate emotions (neurobiologically and behaviorally) from infancy onwards (Thompson 2016). While the early, fundamental function of attachment is to ensure the survival of human infants, emotion regulation may be the primary social function of adult attachment (Mikulincer and Shaver 2016). Furthermore, according to attachment theory, affective states cannot be completely regulated by individuals themselves as “we all are emotional regulators of each other” (p.8; Flores 2011).

Formed through early experiences, internal working models – including mental representations of the self and others – guide future social expectancies and interactions (Bowlby 1982). While individuals with a secure attachment style tend to successfully cope with stressful situations by seeking support or by activating mental representations of support received in the past (Mikulincer and Shaver 2004), individuals with an insecure attachment style attempt to cope by using either hyperactivating (e.g., demanding care, worry, rumination) or deactivating (e.g., distrust, self-reliance) strategies (Shaver and Mikulincer 2005). However, these strategies often fail to regulate emotions and may even amplify distress (Mikulincer and Shaver 2007).

Connected to attachment, a mature personality structure – containing diverse and complex representations – allows a tolerance of ambivalence and contradiction in feelings about self and others (Blatt and Levy 2003). In line with this, the Operationalized Psychodynamic Diagnosis system (e.g., Kessler et al. 2013) states that while a good structural integration implies that an autonomous self can carry out mental conflicts in a mental internal space, this ability diminishes with moderate integration, with a low structural integration finally implying that conflicts can barely be worked out in a mental internal space but have to be primarily worked out in the interpersonal sphere.

### Cognitive reappraisal

The theory that the cognitive appraisal of an adverse situation, as opposed to the situation itself, determines the intensity and quality of an emotional response, has gained wide acceptance since the work of Lazarus and others (e.g., Lazarus 1993). In detail, cognitive reappraisal means the deliberate change in perspective regarding an emotionally evocative situation, thereby re-interpreting its meaning and modulating its emotional impact (Lazarus and Folkman 1984). As this strategy for emotion regulation is thought to be highly effective (Webb et al. 2012), the encouragement of its use is part of several psychotherapeutic approaches. Ideally, and with extensive practice, cognitive reappraisal may become a habitual technique to deal with adverse situations (Hertel 2004) and should exert positive influence on psychological well-being (Gross and John 2003).

As a novel tool in cognitive reappraisal research, the Reappraisal Inventiveness Test (RIT; Weber et al. 2014) assesses individuals’ capacity for generating alternative interpretations for aversive events, which constitutes a crucial development in the science of emotion regulation (e.g., Demaree et al. 2006). Accordingly, instead of focusing on self-reported reappraisal frequency or success (Troy et al. 2010), the RIT is a maximum performance test of the basic cognitive capacity for reappraisal implementation (Papousek et al. 2017; Perchtold, et al. 2018a). This theoretical capacity putatively serves as a direct prerequisite for the ability to effectively implement cognitive reappraisal in everyday life (Weber et al. 2014). While maximum performance tests are widely used in several disciplines, they are a relatively new, yet promising concept for psychotherapy research (Papousek et al. 2017).

### Neural correlates

Attachment bonds are formed in the context of the extensive neural development during the first two years of life (Coan 2016). Repetitive and patterned activations during this development have a large impact on neural organization (Posner and Rothbart 2007). As the pathways linking the amygdala to the hippocampus and regions of the prefrontal cortex are still underdeveloped in neonatal infants (Herschkowitz 2000), the involvement of prefrontal regions in neonatal learning may be limited (Coan 2016). Accordingly, the caregiver may act as a surrogate prefrontal cortex influencing the infant’s neural development and therefore his or her emotion reactivity and regulation throughout life (Gee et al. 2014). As neural changes initiated by early experiences seem to be persistent, this likely leads to an individualized model of the social world and corresponding strategies for engaging or avoiding social stimuli and for regulating emotions. However, during later development, new experiences might reinforce or alter this model (Coan 2016). While secure attachment is associated with less reactivity to distress, insecure attachment seems to be connected to increased neural activation throughout the brain under conditions of distress (e.g., pain or threat; Coan 2016). Specifically, avoidant attachment might be associated with increased prefrontal activation, which indicates an increased focus on emotion regulation due to either a greater emotional burden or regulatory inefficiency. Anxious attachment, on the other hand, has been linked to increased activation in the amygdala, the hippocampus and, especially, the dorsal anterior cingulate cortex (Vrtička et al. 2012).

Regarding emotion regulation, studies focusing on neural correlates of individuals’ capability for cognitive reappraisal are limited (Papousek et al. 2017; Perchtold, Fink et al. 2018). In general, cognitive reappraisal attempts in response to negative stimuli appear to involve the medial frontal cortex, a potential relay station between circuits activated by the cognitive reappraisal of emotional significance and the subcortical circuits activated for emotional responses (Johnstone et al. 2007), as well as the lateral prefrontal cortex in the left hemisphere (Dillon and Pizzagalli 2013). Furthermore, the left ventrolateral prefrontal and orbitofrontal areas seem to be uniquely active during cognitive reappraisal compared to other emotion regulation strategies (Dörfel et al. 2014; Price et al. 2013), which suggests that executive functions strongly influence cognitive reappraisal capacity (Rowland et al. 2013; Weber et al. 2014). The abilities to inhibit negative aspects of a situation and to shift the focus from a negative to a neutral mental set are especially required for cognitive reappraisal (Malooly et al. 2013).

### The present study

In this study, we explored neural correlates of cognitive reappraisal in poly-drug use disorders (PUD) and their association with individuals’ capability to generate cognitive reappraisals for aversive situations as well as other variables relevant for emotion regulation (e.g., attachment and personality structure). While our previous research underlines the presence of impairments in attachment, personality structure and structural neural integrity in PUD (Unterrainer et al. 2017; Unterrainer et al. 2016), the exploratory use of an fMRI paradigm regarding actual cognitive reappraisal capacity in this study should allow further insights into the impaired emotion regulation in substance use disorders. Following research on cognitive reappraisal in other mental disorders (Dillon and Pizzagalli 2013; Johnstone et al. 2007), we hypothesized an attenuated prefrontal activation in patients with substance use disorders during the cognitive reappraisal task.

## Methods and materials

### Participants

The sample consisted of 34 right-handed men: One clinical inpatient group (PUD; *n* = 18) diagnosed for PUD (F19.2) according to ICD-10 (Dilling et al. 1991) and one group of controls from the normal population (*n* = 16) who reported no or very little experience with illegal substances. Control participants had not used psychoactive substances in the last 30 days (except for occasional consumption of alcohol) and had no past or present psychiatric disorder. The study (including all experimental protocols and the informed consent form) was planned and performed in accordance with the Declaration of Helsinki and was approved by the ethics committee of the University of Graz, Austria. Informed consent was obtained from all participants.

### Assessment of cognitive reappraisal

**Fig. 1:**
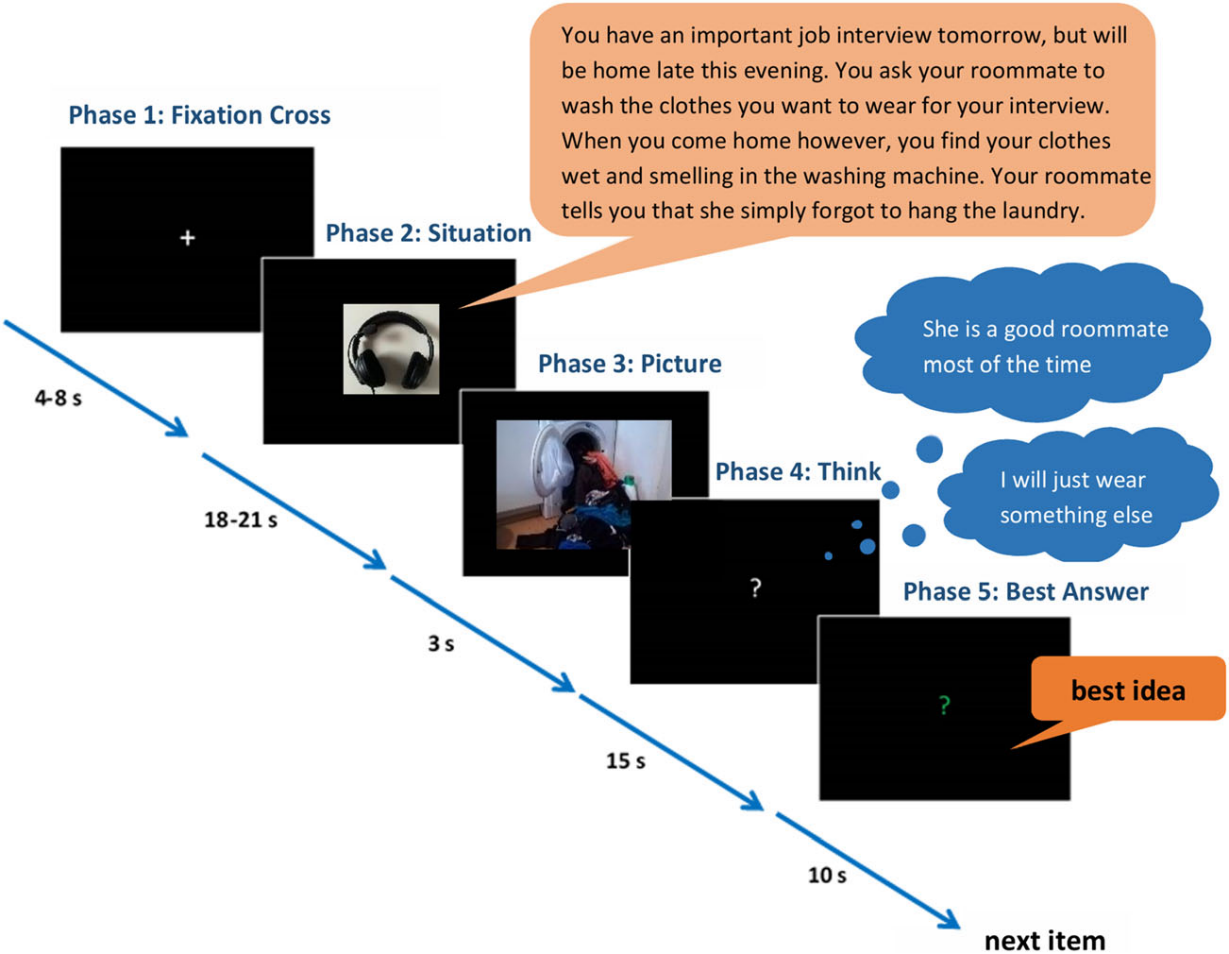
Schematic sequence of an RGT item. A jittered fixation phase (4–8 s) is followed by an audio story of an anger-eliciting event (18–21 s), which is subsequently illustrated by a matching photograph (3 s). This is followed by a thinking phase (15 s), indicated by a white interrogation mark. When the interrogation mark changes its color into green, participants were requested to vocalize their best idea (10 s). (RGT = Reappraisal Generation task)

Cognitive reappraisal capacity was assessed with two tasks, the original Reappraisal Inventiveness Test (RIT; Weber et al. 2014) outside the scanner and a similar Reappraisal Generation Task (RGT; see Perchtold et al. 2018b) during fMRI: In each task, participants were asked to empathize with anger-eliciting situations (where another person willingly or carelessly induces harm) and to come up with different reappraisals to downregulate their anger. See Fig. 1 for an example item.

In line with the scoring procedure of the RIT (Weber et al. 2014), items administered outside the scanner were rated for Fluency (i.e., the total number of generated, non-identical reappraisals) and Flexibility (i.e., the number of categorically different reappraisals). Inter-rater-reliability was excellent (ICC RIT-Fluency = .94, ICC RIT-Flexibility = .91). Subjective anger ratings of the presented scenarios were assessed as well (7-point scale from 0 “not angry at all” to 6 “extremely angry”).

The RGT was specifically designed for an fMRI environment and consisted of 20 vignettes (four original vignettes by Weber et al. 2014; four additional vignettes by Papousek et al. 2017, and twelve new vignettes created for fMRI studies, matching the main characteristics of existing vignettes). All vignettes were designed to match scenarios likely to occur in everyday life to increase the motivation to attentively engage in the task. The RGT allows controlling for adherence to the reappraisal instructions as well as a detailed analysis of reappraisal answers, which is still rare in emotion regulation research (Demaree et al. 2006). A previous study already demonstrated the validity of the RGT in measuring genuine cognitive reappraisal efforts during fMRI in a sample of students (Perchtold, Papousek et al. 2018).

As seen in Fig. 1, each RGT trial started with a jittered fixation interval of 4–8 s. Participants then listened to an audio description of an anger-eliciting situation lasting about 20 s and imagined the situation happening to them. Next, a matching photograph was presented for 3 s, followed by a white question mark displayed for 15 s, during which participants had to think of possible reappraisals to diminish anger. When the question mark changed its color to green, participants had to articulate their best reappraisal within 10 s. The RGT had a total duration of approximately 20 min.

Due to the acoustic item presentation, confounding effects of reading speed and visual text processing could be excluded. All given responses were recorded and transcribed. Two independent raters evaluated the reappraisals in terms of their Effectivity to reduce anger in the respective situation. To avoid potential biases, the raters were unaware of whether the reappraisals were originated from the PUD or the control group. Ratings ranged from 1 (“reappraisal not effective at all”) to 4 (“reappraisal highly effective”). Inter-rater-reliability was satisfactory (ICC RGT-effectivity = .79).

Initially and in line with previous research (Perchtold, Papousek et al. 2018), a control condition to the RGT was planned, which consisted of a divergent thinking task without emotional component. This way, we wanted to ascertain that brain activation patterns of interest were specific to cognitive reappraisal efforts, and not simply due to the presence of cognitive effort or idea generation without affective load. However, after preliminary tests with four PUD inpatients, time in the scanner had to be drastically shortened, as the two complex tasks with a duration of >45 min resulted in restlessness and noncompliance with task instructions. Thus, the control task was dismissed to preserve the overall goal of the study.

#### fMRI data acquisition

Imaging was performed on a 3 T MRI scanner MAGNETOM Skyra (Siemens Medical Systems, Erlangen, Germany) using a 32-channel head coil. Structural images were acquired using a MPRAGE T1-weighted sequence (TR = 1950 ms, TE = 2.89 ms, inversion time = 950 ms, flip angle = 12°, 176 sagittal slices, FOV = 256 × 256 mm). BOLD-sensitive T2*-weighted functional images were acquired using a single shot gradient-echo EPI pulse sequence (TR = 2520 ms, TE = 30 ms, flip angle = 90°, slice thickness = 3.3 mm, 10% distance factor, matrix size = 66 × 66, FoV = 218 mm, 38 axial slices per volume, order descending). The first two volumes were discarded to allow for T1 equilibration effects. In addition to structural and functional images, a dual-echo gradient echo field map (TR = 403 ms, deltaTE = 2.46 ms) was recorded for distortion correction of the acquired EPI images. Head motion was restricted using firm padding that surrounded the head. An MR compatible microphone was utilized to record the verbal responses of the participants (FOMRI-III, Optoacoustics Ltd., Moshav Mazor, Israel). Stimuli were presented with the Software Presentation (Neurobehavioral Systems, Albany, CA).

### Additional behavioral assessment

Habitual use of cognitive reappraisal and suppression were assessed with the Emotion Regulation Questionnaire (ERQ; Abler and Kessler 2009; Gross and John 2003). It contains 10 items, 6 for Reappraisal and 4 for Suppression, rated on a 7-point Likert scale ranging from 1 (strongly disagree) to 7 (strongly agree). Cronbach’s alphas were .74 for Reappraisal and .76 for Suppression (Abler and Kessler 2009).

Attachment styles were assessed with the Adult Attachment Scale (AAS; Schmidt et al. 2004). The AAS consists of three subscales: Anxiety about being rejected or unloved (Anxiety), comfort with closeness (Closeness) and intimacy and comfort depending on others (Dependence). Fifteen items (5 items per sub-scale) are rated on a 5-point Likert scale ranging from 1 (strongly disagree) to 5 (strongly agree). Cronbach’s alphas were .79 for Closeness, .72 for Dependence and .78 for Anxiety (Schmidt et al. 2004).

Impairments in personality structure were assessed with the OPD Structure Questionnaire (OPD-SQ; Ehrenthal et al. 2012). The OPD-SQ assesses the amount of structural disintegration with four dimensions (Kessler et al. 2013) that each comprise a self-related and an object-related subdomain: (1) Perception; (2) Regulation; (3) Communication; (4) Bonding. Theses 8 subscales and a total score of Structural Disintegration are assessed with 95 items rated on a 5-step Likert scale ranging from 0 (totally disagree) to 4 (totally agree). Cronbach’s alphas for the subscales ranged from .72 to .91 (Ehrenthal et al. 2012).

The Wonderlic Personnel Test (WPT; Wonderlic 1999), a rough screening instrument for the assessment of intelligence, requires the processing of disordered sentences, analogies, number series, word and sentence comparisons and geometrical figures within 12 min. The WPT contains 50 items with increasing difficulty. The total score is generated from the number of correct responses.

The Brief Symptom Inventory (BSI-18; Franke et al. 2011) assesses the amount of psychiatric burden (Somatization, Depression, and Anxiety) for the preceding seven days. The 18 items (6 per scale) are rated on a 5-point Likert scale ranging from 0 (absolutely not) to 4 (very strong). A Global Severity Index (GSI) can be generated as a total score. Cronbach’s alpha was at least at .79 for the sub-dimensions and .91 for the GSI (Franke et al. 2011).

### Statistical analysis

#### fMRI data analysis

Functional MRI data analysis was performed using SPM 12 software (v6906; Wellcome Department of Imaging Neuroscience, London, UK), which ran in a MATLAB 2015b environment (Mathworks Inc., Natick MA, USA). Images were corrected for geometric distortions by the use of the FieldMap toolbox (Hutton et al. 2002). All fMRI data were preprocessed using Data Processing Assistant for Resting-State fMRI (DPARSF, v4.1_160415), which is part of DPABI (Yan et al. 2016). Images were realigned and unwarped, slice-timed corrected and then coregistered to the high-resolution structural image, which was segmented with the DARTEL toolbox of SPM. All functional datasets were then spatially normalized into the standard Montreal Neurological Institute (MNI) space and smoothed using a 9 mm FWHM Gaussian spatial kernel.

Voxel-wise, whole brain comparisons were used for all analyses. First level analyses were performed by computing linear t-contrasts (groups vs. implicit baseline consisting of the fixation cross and between groups) for the reappraisal generation interval (15 s) for each participant individually, which were then entered into random effects one-sample t-tests. Six motion parameters, white matter signal, CSF signal, and global signal were included in the model as regressors of no interest. A preliminary analysis of motion parameters yielded no significant differences between PUD and controls (all p’s > .11). To depict a general reappraisal-related brain activation pattern, the overlap in brain activation between PUD and the control group was examined by contrasting the reappraisal generation phase in both groups against the implicit baseline (consisting of the fixation cross) (Poline et al. 2003). In addition, a conjunction analysis was computed to identify voxels that were significantly activated in both groups during performance of the RGT (e.g., Nichols et al. 2005). A two-sample t-test was then used to explore group differences in brain activation between PUD and the control group during reappraisal generation. All task-related effects are corrected for multiple comparisons at the voxel level by means of the conservative FWE (family wise error) procedure implemented in SPM 12. Thus, activations passing a height threshold of *p* < .05 (for contrasts against implicit baseline and for contrasts between the groups) were considered significant.

#### Behavioral data analysis

For group comparisons, one-way analyses of variance were conducted. To allow a better evaluation of the results, effect sizes were generated. In addition, Pearson’s correlation statistics were applied to investigate the relationships between parameters in PUD. In consideration of the limited sample size and the exploratory nature of the study, alpha was set to *p* < .05. Behavioral analyses were performed using the software IBM SPSS Statistics 25.

## Results

### Demographics and behavioral characteristics

PUD had been in inpatient treatment for a mean time of 24 weeks (*SD* = 17.15) at the time of the study. Six PUD were in maintenance therapy, while 12 were drug-free. PUD in maintenance therapy received Levo-Methasan® or Substitol®, daily doses varied between inpatients. Several inpatients received psychopharmacological medications (anxiolytic: *n* = 2; antipsychotic: *n* = 4; antidepressant: *n* = 5; others: *n* = 5). As detailed in Table 1, PUD were older (*eta*^*2*^ *= .*16; *p* = .021) than the control group while reporting fewer years of education (*eta*^*2*^ *= .*26; *p* = .002). PUD also scored significantly lower on the WPT screening measure for intelligence, indicating lower cognitive abilities (*eta*^*2*^ *= .*62; *p* < .001).

**Table 1:** Group differences (ANOVAs) in demographic variables, cognitive reappraisal capacity and behavioral measures

	Controls (*n* = 16)		PUD (*n* = 18)				
Measure	*M*	*SD*	*M*	*SD*	F_(*1,34*)_	*p*	*eta*^*2*^
Age (years)	24.50	3.76	28.11	4.78	5.89*	.021	.16
Education (years)	13.13	2.42	10.83	1.51	11.28**	.002	.26
WPT	28.56	4.27	15.61	5.85	52.73**	<.001	.62
OPD-SQ							
Self-perception	.67	.43	1.68	.70	24.61**	<.001	.44
Object perception	.99	.42	2.03	.48	44.77**	<.001	.58
Self-regulation	.92	.48	1.86	.60	25.34**	<.001	.44
Regulation of relationships	1.18	.57	2.01	.68	14.78**	.001	.32
Internal communication	.72	.33	1.75	.59	38.05**	<.001	.54
External communication	1.28	.50	1.81	.53	9.04**	.005	.22
Attachment to internal objects	1.13	.71	2.07	.67	16.00**	<.001	.33
Attachment to external objects	1.47	.60	2.18	.48	14.61**	.001	.31
Total	1.04	.40	1.92	.44	36.37**	<.001	.53
AAS							
Closeness	2.98	.89	1.94	.89	10.53**	.003	.25
Dependence	3.53	.53	2.41	.67	28.15**	<.001	.47
Anxiety	1.96	.94	2.42	.82	2.32	.137	.07
BSI							
Global Severity Index	9.19	6.16	16.72	10.64	6.17*	.018	.16
ERQ							
Suppression	2.83	.93	3.88	1.84	4.21*	.048	.12
Reappraisal	5.07	1.06	4.24	1.00	5.51*	.025	.15
RIT							
Anger	3.17	.83	3.94	1.07	5.43*	.026	.15
Fluency	4.41	1.02	2.94	1.24	13.94**	.001	.30
Flexibility	4.19	.97	2.86	1.12	13.40**	.001	.30
RGT							
Effectivity	2.52	.32	2.16	.43	7.43*	.010	.19

*Note.* CR = Cognitive Reappraisal, PUD = Poly-drug users, WPT = Wonderlic Personnel Test, OPD-SQ = OPD Structure Questionnaire, BSI = Brief Symptom Inventory, AAS = Adult Attachment Scale, ERQ = Emotion Regulation Questionnaire, RIT = Reappraisal Inventiveness Test, RGT = Reappraisal Generation Task

Furthermore, we found several differences with generally large (*eta*^*2*^ > .14) effect sizes – as defined by Cohen (1988) – between PUD and the control group (see Table 1): Regarding personality structure, PUD showed higher levels of structural disintegration in all self and object related subscales (all *eta*^*2*^ > .30; *p* < .01) with differences being especially pronounced in Object perception (*eta*^*2*^ = .58, *p* < .001) and Internal communication (*eta*^*2*^ = .54, *p* < .001). Regarding attachment, PUD showed lower levels of Closeness (*eta* ^*2*^ = .25; *p* = .003) and Dependence (*eta*^*2*^ = .47; *p* < 001) while they did not differ from controls regarding Anxiety (*p =* .137). This was paralleled by a higher level of the GSI in PUD (*eta*^*2*^ = .16; *p =* .018).

Regarding emotion regulation, PUD reported a more frequent use of Suppression than the control group (*eta*^*2*^ = .12; *p* = .048) but a less frequent use of Reappraisal (*eta*^*2*^ = .15; *p* = .025).

### Cognitive reappraisal

Regarding cognitive reappraisal capacity (see Table 1), PUD demonstrated lower Fluency and Flexibility of ideas (both *eta*^*2*^ = .30; both *p* = .001) and reported higher Anger with the depicted reappraisal situations (*eta*^*2*^ = .15; *p* = .026) than the control group. During fMRI, their reappraisal ideas were rated as less effective than those generated by the control group (*eta*^*2*^ = .19; *p* = .010). Group differences in Fluency and Flexibility remained virtually unchanged when controlling for scores on the WPT screening for cognitive abilities. However, controlling for WPT scores rendered group differences in other-rated Effectivity of reappraisal ideas (*eta2* = .05; *p* = .195) and self-reported Anger (*eta2* < .001; *p* = .960) non-significant.

**Fig. 2:**
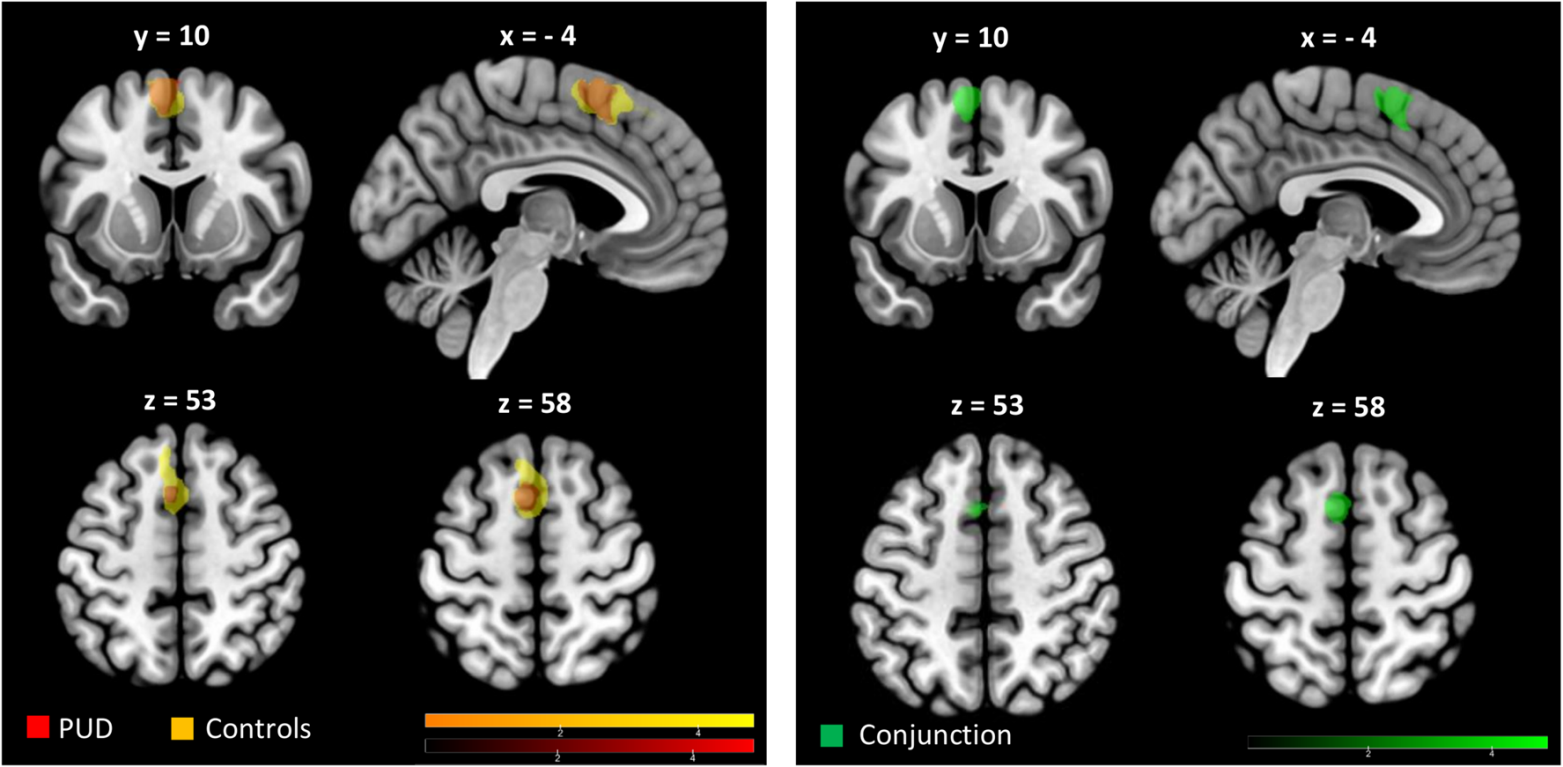
Overlap in brain activation between PUD and the control group supplemented by a conjunction analysis. Whole brain analysis (T maps) of brain activation during RGT for PUD and the control group relative to implicit baseline (all effects voxel-wise *p* < .05 FWE corrected, k > 20). Both PUD (red colors) and the control group (yellow colors) showed brain activation predominantly in the left frontal cortex including superior, middle, and inferior frontal gyri as well as supplemental motor areas. Green colors indicate the results of the conjunction analysis of RGT activation in both PUD and the control group, denoting activation in the left superior and middle frontal gyrus as well as supplemental motor areas (CG = control group, PUD = Poly-drug users, RGT = Reappraisal Generation Task)

The reappraisal-related neural activation pattern during the RGT observed for both PUD and the control group was remarkably similar (see Fig. 2) and revealed a network predominantly in the left frontal cortex including the inferior, superior, and middle frontal gyri, as well as supplemental motor areas. An additional conjunction analysis on voxels significantly activated in both groups revealed in more detail that PUD and the control group activated the left middle and superior frontal gyri, as well as the left supplementary motor cortex during cognitive reappraisal of aversive events (see Fig. 2). Coordinates of regional peak activations for the conjunction analysis are shown in Table 2. No group differences in neural activation were found.

**Table 2:** Overview of significant activation clusters for the conjunction analysis of reappraisal-related brain activation shared by PUD and CG

Location	MNI peak coordinate	k	t-max
*Conjunction PUD and CG*			
L SMA L sup frontal G, L mid frontal G	-6, 9, 63	101	7.92

*Note.* Voxelwise *p* < .05 FWE corrected, *k* > 20; Coordinates are reported in MNI space as given by SPM 12 and correspond only approximately to the Talairach and Tournoux (1988) space. Anatomical labels are based on the AAL (automated anatomical labeling) atlas (Tzourio-Mazoyer et al. 2002). Location, MNI peak coordinates, cluster size k and maximum t-value of the significantly activated clusters. The first label represents the location of the peak activation; additional labels denote further brain areas covered to at least 20% by the activation cluster. (CG = Control Group, PUD = Poly-drug users*,* L = left hemisphere, mid = middle, G = gyrus, SMA = supplemental motor areas)

### Intercorrelations in PUD

Table 3 depicts correlations between attachment, personality structure and emotion regulation in PUD: While more structural disintegration was connected to lower levels of secure attachment (Dependence: *r* = −.54; *p* = .021; Closeness: *r* = −.68; *p* = .002), higher levels of insecure attachment were in turn connected to less Fluency (*r* = −.49; *p* = .037) and Flexibility of ideas (*r* = −.48; *p* = .042) in the RIT. Higher levels of induced Anger in the RIT were connected to a higher GSI (*r* = .68; *p* = .002). Only self-reported use of Suppression, but not the use of Reappraisal, showed a negative connection to treatment duration (Suppression: *r* = −.61; *p =* .007; Reappraisal: *r* = .18; *p* = .482).

**Table 3:** Intercorrelations between cognitive reappraisal capacity and behavioural characteristics in PUD

	1.	2.	3.	4.	5.	6.	7.	8.	9.	10.	11.	12.	13.
OPD-SQ													
1. Total	–	−.68**	−.54*	.29	.08	.24	.39	−.10	−.05	.26	.46	.06	.22
AAS													
2. Closeness		–	.70**	.07	−.05	−.05	−.02	−.15	−.19	−.13	−.17	−.34	−.36
3. Dependence			–	.14	.03	−.27	.25	−.24	−.17	−.29	.09	−.18	−.20
4. Anxiety				–	−.13	.31	.36	−.49*	−.48*	.25	.13	−.23	.26
ERQ													
5. Suppression					–	−.29	.14	−.12	.16	−.01	.02	.22	−.61**
6. Reappraisal						–	−.04	−.23	−.18	.14	−.29	.07	.18
RIT													
7. Anger							–	−.39	−.23	−.24	.68**	−.42	.13
8. Fluency								–	.84**	.31	−.19	−.22	.12
9. Flexibility									–	.35	−.09	−.04	−.16
RGT													
10. Effectivity										–	−.06	.07	−.14
11. GSI											–	−.27	−.04
12. WPT												–	−.22
13. Treatment													–

*Notes*. * *p* < .05, ** *p* < .01, PUD = Poly-drug users, OPD-SQ = OPD Structure Questionnaire, AAS = Adult Attachment Scale, ERQ = Emotion Regulation Questionnaire, RIT = Reappraisal Inventiveness Test, RGT = Reappraisal Generation Task, GSI = Global Severity Index (BSI-18), WPT = Wonderlic Personnel Test, Treatment = Duration of current inpatient treatment at the time of the study (in weeks)

## Discussion

In this study we focused on several parameters relevant to treatment of substance use disorders (Flores 2011). Specifically, we investigated the capacity for cognitive reappraisal as well as other variables linked to emotion regulation (e.g., attachment and personality structure) in PUD since studies on specific emotion regulation strategies and their neural correlates in substance use disorders are still relatively sparse (Aldao et al. 2010).

Our results not only confirm our previous findings of insecure attachment and impaired personality structure in substance use disorders (Hiebler-Ragger et al. 2016; Unterrainer et al. 2016) but also underline the prevalence of impaired emotion regulation abilities in PUD. As a central finding, PUD inpatients displayed a significantly lower capacity for inventing cognitive reappraisals to deal with anger-eliciting events compared to the control group. This was evident in less fluent and less flexible cognitive reappraisal generation as well as higher subjective anger induced by the situations in PUD, paralleled by lower externally rated effectivity of reappraisal ideas generated by PUD during fMRI. Interestingly however, these marked behavioral differences to the control group were not mirrored on a neural level. Here, relatively similar activation patterns during cognitive reappraisal efforts emerged, with no significant differences between PUD and the control group. In line with research on cognitive reappraisal in other mental disorders (Dillon and Pizzagalli 2013; Johnstone et al. 2007), we had expected an attenuated prefrontal activation in PUD during the cognitive reappraisal task. This assumption could not be confirmed.

Regarding attachment and personality structure, our results not only underline the connection between these concepts and their relevance in substance use disorders, but also their association to emotion regulation. In detail, PUD seem to have internalized negative/maladaptive self-related and object-related mental representations in all areas of personality structure defined by the OPD system (Kessler et al. 2013). These mental representations likely impair their ability for social interaction and emotion regulation: For example, impairments in self-perception include problems with affect differentiation, impairments in self-regulation and the regulation of relationships include problems in affect tolerance as well as impulse control, while impairments in internal and external communication include problems in experiencing and communication affect. Lastly, impairments regarding the attachment to internal and external objects include less variability of attachment patterns as well as problems with accepting help (Kessler et al. 2013). Similarly, the psychodynamic self-medication model of substance use disorders formulated by Khantzian and colleagues (e.g., Khantzian 1997) includes two critical parameters (disordered self-care and disordered emotions) as well as two contributory parameters (disordered relationships and disordered self-esteem) that lead individuals to use psychotropic substances to cope with a dysregulated affective state. This supports the notion that affective states cannot be completely regulated without aid (Flores 2011) and that emotion regulation may be the most important function of adult attachment (Mikulincer and Shaver 2016).

Our results regarding induced anger in PUD are in line with previous research indicating higher levels of primary negative emotions in PUD compared to the control group (Unterrainer et al. 2017). As increased negative emotional states also seem to be associated with a higher rate of relapse, emotion regulation is of integral importance for treatment of substance use disorders (Larimer et al. 1999). Notably, more anger also seems to be connected to less religious/spiritual well-being (Hiebler-Ragger et al. 2018). Correspondingly, a low amount of religious/spiritual well-being was observed in patients with substance use disorders (Unterrainer et al. 2013). This is of special interest, as spirituality is considered a helpful factor in treatment of substance use disorders (Davis and Panksepp 2011) that may support secure attachment experiences and consequently increase the ability to deal with adversity (Unterrainer et al. 2013). Most prominently, the therapeutic community (De Leon 2000) – a long-term, caregiving inpatient treatment concept – focuses on facilitating corrective emotional experiences that allow a post-maturation of previously insecure attachment patterns (Flores 2001).

Much like the activation of attachment strategies relies on the subjective appraisal of a situation as distressing (Bowlby 1982), the model of stress and coping by Lazarus and Folkman (1984) highlights the critical role of appraisal in the regulation of distress. While we did not find the expected differences between PUD and the control group regarding neural activation during cognitive reappraisal, the overall activation pattern of the two groups fits almost perfectly with previous findings, that reappraisal efforts elicit activation in the left frontal cortex, particularly in the left superior and middle frontal gyri and left supplemental motor areas (Perchtold, Papousek et al. 2018). This not only corroborates the importance of the frontal cortex and consequently of executive functions in cognitive reappraisal of critical situations (Rowland et al. 2013; Weber et al. 2014). Together with our analyses of reappraisal answers, it also suggests genuine reappraisal efforts in both groups. In this regard, it is possible that the control group exhibited a more efficient pattern of neural activation during reappraisal efforts in order to perform adequately in the task, while for PUD, the same level of brain activation resulted in significantly poorer task performance. Alternatively, the discrepancy between behavioral and neural results might suggest a third variable linking these two levels. However, other imaging modalities will be needed to test these assumptions. Importantly, our previous research indicated that white matter is especially impaired in PUD (Unterrainer et al. 2017; Unterrainer et al. 2016). Thus, cognitive reappraisal efforts may lead to the required activation in grey matter structures in PUD but the impaired white matter may inhibit the required interaction between relevant neural circuits resulting in poorer cognitive reappraisal capacity. Furthermore, as rather maladaptive strategies associated with different types of insecure attachment– hyperactivating (e.g., demanding care, worry) in anxious attachment and deactivating (e.g., distrust, self-reliance) strategies in avoidant attachment (Shaver and Mikulincer 2005) – also seem to be associated with different neural activation patterns during emotion processing (Ma et al. 2017) and regulation (Vrtička et al. 2012), the combination of these patterns might mask their difference to activations in individuals with secure attachment (i.e., the control group). Notably, secure attachment does not eliminate the experience of anger towards others: While insecure attachment likely leads to an “anger of despair” based on a distrust of relationships and little expectations towards others, secure attachment likely facilitates an “anger of hope” based on the willingness to invest in relationships (Mikulincer 1998). Therefore individuals with secure attachment might feel more positive emotions along with their anger, including more optimistic expectations towards the other person and the possibility to resolve a situation (Mikulincer 1998).

Note again that the original plan of this study was to implement a control condition of divergent thinking without affective components (see Perchtold, Papousek et al. 2018). This way we intended to assess brain activation during the RGT specifically related to participants’ engagement in the emotion regulation strategy of cognitive reappraisal and not to general idea generation processes without affective component. Administering two complex cognitive fMRI tasks, however, was ultimately not feasible with our PUD inpatients, since it resulted in unrest, severe concentration difficulties, and less compliance with task instructions. But critically, the overall pattern of brain activation during the RGT was very similar to the results of a previous fMRI study (Perchtold, Papousek et al. 2018), generally corroborating the reliability and validity of the obtained patterns of findings in this study.

Interestingly, we only found associations between the use of suppression – but not reappraisal – and treatment duration in the therapeutic community of this study. This underlines the necessity to focus future research on several emotion regulation strategies and their possible importance for clinical treatment of substance use disorders. Furthermore, the different possible mechanisms of cognitive reappraisal in respective patients will have to be explored, as reappraisal might target the meaning or the self-relevance of a potentially emotion-eliciting experience to decrease or increase positive or negative emotions (Gross 2015). In addition, there is some indication that cognitive reappraisal might only be adaptive in dealing with uncontrollable stress, where self-regulation is the only option, but not controllable stress, where the situation can be changed (Troy et al. 2013).

As regards the obtained group differences in reappraisal parameters, it is important to note that only the differences in reappraisal Fluency and Flexibility persisted when controlling for scores on the WPT screening of cognitive abilities. It may thus be suggested that differences in cognitive abilities play a larger role for subjective anger experience and rated effectiveness of reappraisal ideas than PUD itself, which is not surprising, given that in the present study, reappraisal generation was operationalized as a cognitive capacity (see Weber et al. 2014). Nonetheless, future studies with larger samples should include more comprehensive intelligence batteries or specific cognitive tests to clarify the role of more general cognitive abilities for cognitive reappraisal in PUD.

While the applied reappraisal tasks have several advantages over other approaches (e.g., the control for adherence to the instructions), the ad hoc generation of several different reappraisals for one situation (RIT) is still a relatively new concept for clinical research (Papousek et al. 2017). Therefore, the relevance of reappraisal inventiveness in clinical groups will have to be explored in more detail in future studies, which might also benefit from implementing pre- and post-reappraisal ratings to assess how much their reappraisal attempts reduced their emotional experience (e.g., anger) with a specific scenario. Furthermore, while we sought to pinpoint neural correlates of cognitive reappraisal in PUD using fMRI, future research might expand this approach by using different parameters. EEG in particular may enable a more time-sensitive analysis of different stages in the reappraisal process (Kalisch 2009). A combination of fMRI and EEG as well as the consideration of personality factors (e.g., neuroticism) and sex differences might also prove interesting, as recent studies on alcohol-related craving (Huang et al. 2018) and emotional face processing (Klamer et al. 2017) suggest. In addition, future studies should explore possible neuroplasticity related to the influence of substance disorder treatment on emotion regulation abilities.

Lastly, we did not differentiate between various forms of insecure attachment (e.g., anxious and avoidant attachment) and impaired personality structure (e.g., different levels of structural integration) that are thought to differentially impact an individuals preferred form of dealing with distress (Shaver and Mikulincer 2007). Overall, studies with larger samples will be needed to allow a more thorough consideration of the complexity of substance use disorders and to consequently enhance the interpretation of result, for example by including co-morbid psychiatric disorders, drug of choice and addiction severity. From a developmental perspective, attention-deficit hyperactivity disorder (ADHD) may be of particular interest in this regard, as it has also been linked to emotional dysregulation, difficult temperament, and attachment issues, and is thus known to have a high co-morbidity with other psychiatric disorders, including substance use disorders (Gallo and Posner 2016; Posner et al. 2020). It is therefore possible that our results are influenced by neurobiological mechanisms that represent a common risk factor for several psychiatric disorders, which should be clarified in future investigations.

## Conclusions

While more research on cognitive reappraisal and its neural correlates in PUD is strongly recommended, our results indicate that PUD is linked to impaired cognitive reappraisal capacity in dealing with anger-eliciting events, which is matched by impairments in personality structure and attachment; yet in our study, was not mirrored in different brain activation patterns during cognitive reappraisal efforts compared to controls. In light of these findings, neuro-scientifically informed approaches that consider habit and ability aspects of emotion regulation – including attachment and personality structure – may enrich the ways of relating to patients treated for substance use disorders (Clark et al. 2012).

## Data Availability

The datasets generated during and/or analyzed during the current study are available from the corresponding author on reasonable request.
